# Arresting of miR-186 and releasing of H19 by *DDX43* facilitate tumorigenesis and CML progression

**DOI:** 10.1038/s41388-018-0146-y

**Published:** 2018-02-16

**Authors:** J. Lin, J.-C. Ma, J. Yang, J.-Y. Yin, X.-X. Chen, H. Guo, X.-M. Wen, T.-J. Zhang, W. Qian, J. Qian, Z.-Q. Deng

**Affiliations:** 1 Department of Central Lab, Affiliated People’s Hospital of Jiangsu University, Zhenjiang, Jiangsu China; 2 The Key Laboratory of Precision Diagnosis and Treatment in Hematological Malignancies of Zhenjiang City, Zhenjiang, Jiangsu China; 3 Department of Hematology, Affiliated People’s Hospital of Jiangsu University, Zhenjiang, Jiangsu China; 4 Department of Otolaryngology, Affiliated People’s Hospital of Jiangsu University, Zhenjiang, Jiangsu China

## Abstract

Cancer-testis (CT) antigens, rarely in normal tissues except testis, are expressed in many tumor types. In recent years, *DDX43* has been shown to be expressed in several malignancies. However, the role of *DDX43* during tumorigenesis is not well established. In the present study, we explored the function of *DDX43* in chronic myeloid leukemia (CML). We found that *DDX43* overexpression in CML cell lines enhanced survival and colony formation, inhibited cell apoptosis, promoted tumorigenesis, and CML progression. In contrast, silencing of *DDX43* inhibited cell survival and tumorigenesis. Upregulated H19 and downregulated miR-186 were identified in *DDX43*-transfected cells. Furthermore, we demonstrated that miR-186 targeted *DDX43*, and overexpressed miR-186 increased apoptosis and decreased cell survival. We also showed that *DDX43* regulated the expression of H19 through demethylation and silencing H19 inhibited cell survival. Taken together, these results indicate that *DDX43* provides critical support to the progression of CML by enhancing cell survival, colony formation, and inhibiting cell apoptosis, thereby implicating *DDX43* as a potential therapeutic target in CML.

## Introduction

Chronic myeloid leukemia (CML), present of a distinct translocation t(9;22)(q34;q11), is a malignant myeloproliferative disorder arising from pluripotent stem cells [1, 2]. The t(9;22) rearrangement generates the *BCR-ABL1* fusion gene [3, 4]. According to the natural course of disease, three stages of CML are clinically recognized: an initial chronic phase (CP), then an accelerated phase (AP), and a terminal blast crisis (BC) [1]. During this course, gene mutations and chromosomal abnormalities are involved in the progression of CML [5–7]. However, the mechanisms underlying the evolution need to be further explored. Our team has reported that low methylation of *DDX43* may be related to the development of CML [8].

*DDX43* (also known as HAGE) is initially found as a cancer/testis antigen overexpressed in many solid tumors but absent in normal tissues except testis [9], which indicates this gene has highly tissue-specific expression. These important characteristics make it become a hot spot in the tumor biological treatment and supposed to be an ideal target molecule for cancer therapy.

In hematologic malignancies, *DDX43* is overexpressed in acute myeloid leukemia (AML), CML, multiple myeloma and a variety of malignant cell lines derived from B or T lymphocytic cells [8, 10–13]. Our group found that *DDX43* was highly expressed and associated with the hypomethylation of its promoter in AML and CML [8, 10]. In this study we found that *DDX43* promoted tumorigenesis and CML progression by enhancing cell adhesion, survival, and colony formation.

H19 is a long chain non-coding RNA and its length is about 2.3 kb. Abnormal H19 expression, identified in many solid tumors, such as bladder cancer, breast cancer, liver cancer, lung cancer, plays an important role in tumor growth [14–20]. Several studies have reported that miR-186 influences tumor development by inhibiting tumor cell growth and regulating cell cycle, as a tumor suppressor miRNA [21–23]. We had reported that low expression of miR-186 was associated with poor prognosis in AML [24].

In the present study, we discovered that *DDX43* overexpression in CML cell lines enhanced survival and colony formation, inhibited cell apoptosis, promoted tumorigenesis, and CML progression. In contrast, silencing of *DDX43* inhibited cell survival and tumorigenesis. Furthermore, we demonstrated that miR-186 targeted *DDX43*, and *DDX43* regulated H19 expression through demethylation. All of these play a very important role in the progression of CML.

## Results

### DDX43 affects apoptosis and survival of leukemia cells

**Fig. 1:**
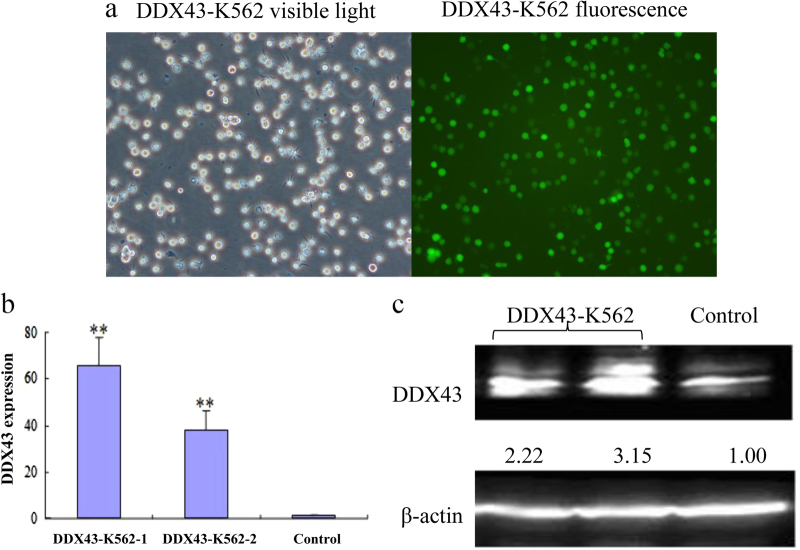
Expression of *DDX43* in CML cells. **a** The expression of *DDX43* from *DDX43*-K562 cells under a fluorescent microscope (×100). **b** Results from RQ-PCR of control cells and two *DDX43*-K562 clones. **c** Western blot of *DDX43*-K562 and control cells. **P < 0.01. Error bars indicate the standard deviation (SD) (*n* = 3)

To explore the role of *DDX43*, a construct expressing *DDX43* was generated and stably expressed in leukemia line K562 (*DDX43*-K562). *DDX43* mRNA and protein were detected using real-time quantitative PCR (RQ-PCR) and western blot, respectively. *DDX43* level significantly increased in *DDX43*-transfected cell lines compared with the mock transfection (Fig. 1).

**Fig. 2:**
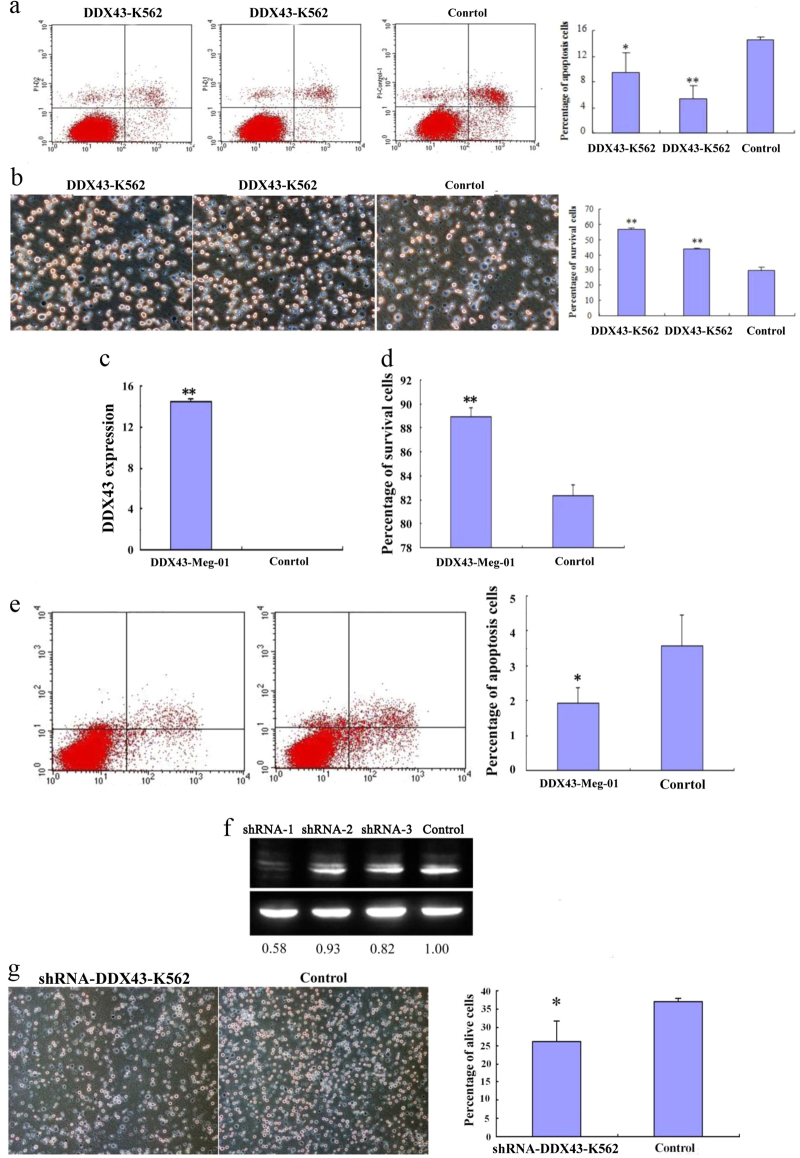
K562 cell apoptosis and survival affected by *DDX43*. **a** *DDX43*-K562 or control cells were cultured for 48 h. Expression of *DDX43* inhibited cell apoptosis. **b** *DDX43*-K562 or control cells were cultured in serum-free conditions. *DDX43* promoted the survival of transfected cells. **c** Expression of *DDX43* in *DDX43*-Meg-01 or control cells were detected by RQ-PCR. **d**, **e** Cell survival and apoptosis were assayed in Meg-01. **f** Expression of *DDX43* in *DDX43* shRNA or control vector transfected *DDX43*-K562 cells was detected by western blot. Lanes 1–3: *DDX43*-K562 transfected with three shRNAs; Lane 4: *DDX43*-K562 transfected with a negative control. ShRNA-1 significantly decreased *DDX43* expression. **g** Cell survival was assayed in *DDX43* shRNA-1-transfected K562. Error bars indicate SD (*n* = 3). *P < 0.05; **P < 0.01

Apoptosis was analyzed for *DDX43*-K562 cells and a control vector transfected K562 cells in serum-free culture conditions for 48 h. Cells were subjected to staining, followed by flow cytometry analysis. *DDX43*-K562 showed less apoptosis than the control cells (Fig. 2a). Survival was also analyzed for these cells cultured in serum-free conditions for 3 days. *DDX43*-K562 cells showed significantly increased survival as compared with control (Fig. 2b). Cell apoptosis and survival were also assayed for the leukemia cell line Meg-01 (Fig. 2c–e), and similar results were observed, confirming that *DDX43* increased survival and reduced apoptosis of these cells. To further confirm the functions of *DDX43*, we used three shRNAs to silence *DDX43* expression in *DDX43*-K562 cells, and significantly downregulated expression of *DDX43* was observed in shRNA-1-transfected *DDX43*-K562 (Fig. 2f). The effects of shRNA on *DDX43*-K562 survival were also tested. Transfection of the shRNA-*DDX43* decreased survival as compared with control (Fig. 2g).

### DDX43 facilitates colony formation and tumorigenesis

**Fig. 3:**
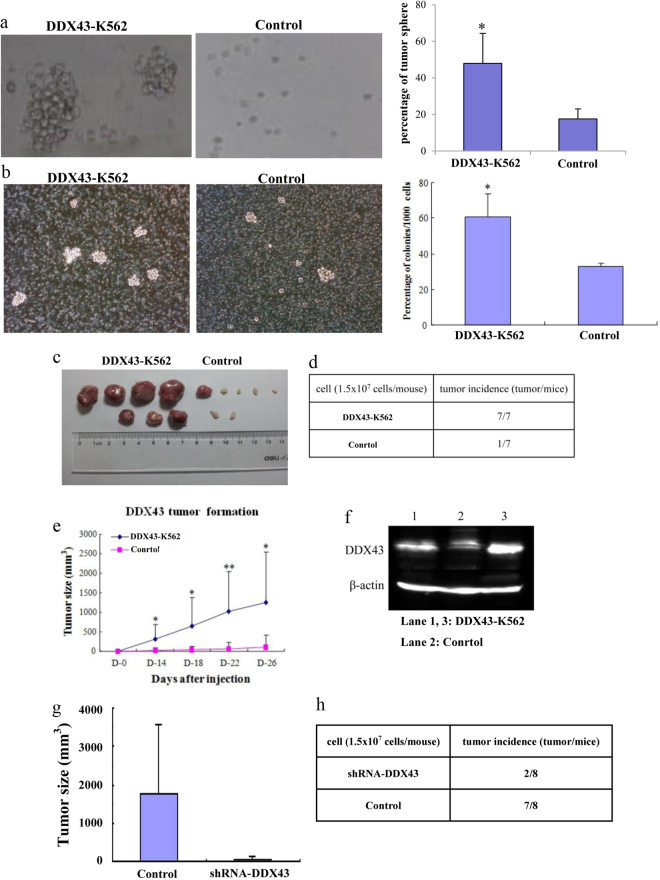
*DDX43* overexpression affected clone formation and tumor formation. **a** Firstly, 2% agarose was plated into six-well plates, and *DDX43*-K562 or control cells were mixed with 0.3% low melting agarose, respectively. More and larger colonies were observed in the *DDX43*-K562 group than in the control group after 4 weeks. **b** K562 cells stably transfected with *DDX43* or the control vector were plated in 1% methylcellulose with 10% FBS. **c**, **d** *DDX43*- or mock-transfected K562 cells were inoculated into CD1 strain nude mice subcutaneously. Mice were photographed and killed after 4 weeks of injection. **e** We measured tumor sizes and the tumor growth curves were drawn. Data showed the mean ± SD from three experiments. **f** DDX43 protein detected by western blot in tumors from mice inoculated with *DDX43*-K562 or control cells. **g** K562 cells transfected with *DDX43* shRNA-1 or a control vector were inoculated into CD1 strain nude mice subcutaneously. Mice were photographed and killed after 4 weeks of injection. Tumor sizes were measured. **h** Tumor incidence from K562 cells transfected with *DDX43* shRNA-1 or a control vector. *P < 0.05; **P < 0.01

To further identify the role of *DDX43* in tumorigenesis, K562 cells were inoculated into soft agarose. Colonies were numbered and photographed after 4 weeks. More and larger colonies were found in *DDX43* group, compared with control (Fig. 3a). Clone formation in methylcellulose was also tested. *DDX43*-K562 and vector-K562 cells were plated in 1% methylcellulose with 10% fetal bovine serum (FBS). The colonies formed by the cells transfected with *DDX43* were more and larger than control cells (Fig. 3b). These results suggested that *DDX43* could promote colony formation.

To further confirm the role of *DDX43* on tumorigenesis in vivo, *DDX43*-K562 or vector-K562 cells were injected subcutaneously into CD1 nude mice. The growth of tumor and the size of tumor were monitored every 4 days. The mice inoculated with *DDX43*-K562 cells developed more and larger tumors than those with control cells (Fig. 3c–e). *DDX43* expression in tumor was detected by western blot, which showed that *DDX43* was highly expressed in the *DDX43*-K562 group (Fig. 3f). *DDX43*-K562 cells transfected with the control vector or shRNA-1 were inoculated into nude mice subcutaneously. Mice were killed after 4 weeks and tumor sizes were measured. We found less and smaller tumors in the *DDX43* shRNA-1-transfected group, compared with the control vector-transfected group (Fig. 3g, h). All mentioned above confirmed the role of *DDX43* in tumorigenesis.

### DDX43 promotes CML progression

In a previous study, the expression of *DDX43* transcript was assayed in bone marrow samples from CML patients [25]. *DDX43* overexpression was identified more frequently in patients at AP/BC stages (4/4, 100%) than those at CP stage (5/22, 23%). These results suggested that *DDX43* expression was relevant to the progression of CML [25].

**Fig. 4:**
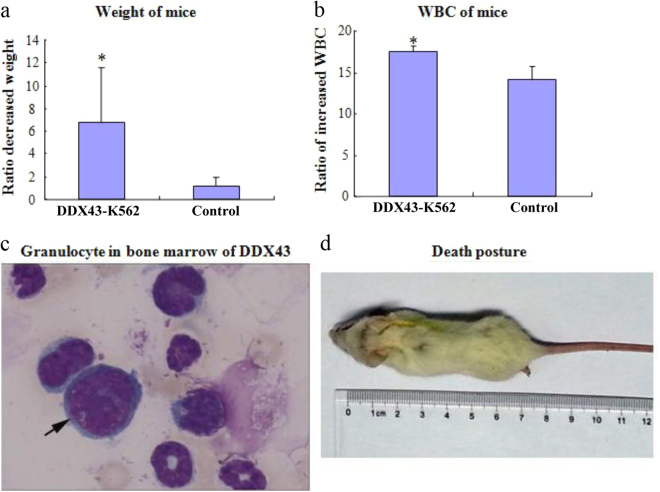
CML progression affected by *DDX43* overexpression. *DDX43*-K562 or control cells were injected via tail vein into SCID mice. **a** The ratio of decreased weight of SCID mice injected with K562 transfected cells. Data shown were mean ± SD from three experiments. **b** The ratio of increased WBC of SCID mice injected with K562 transfected cells. **c** Mice were killed after 4 weeks of injection and blasts in bone marrow of SCID mice injected with *DDX43*-K562 transfected cells were detected with Wright−Giemsa stain. **d** Mice were photographed and killed after 4 weeks of injection. *P < 0.05. Error bars indicate SD (*n = *5)

To further test the role of *DDX43* in CML progression, *DDX43*-K562 or vector-K562 cells were injected via tail vein into SCID mice. We found that the ratio of weight loss was higher in SCID mice injected with *DDX43*-transfected cells than control (Fig. 4a). The ratio of white blood cell (WBC) increase was higher in mice injected with *DDX43*-transfected cells than control (Fig. 4b). Mice were photographed (Fig. 4d) and killed after 4 weeks of injection. Bone marrow was separated and stained with Wright−Giemsa; myeloblasts were easily found in the group injected with *DDX43*-transfected cells, but not in the control group (Fig. 4c). These results suggested the role of *DDX43* in CML progression.

### Release of H19 by demethylation mediates DDX43 function

**Fig. 5:**
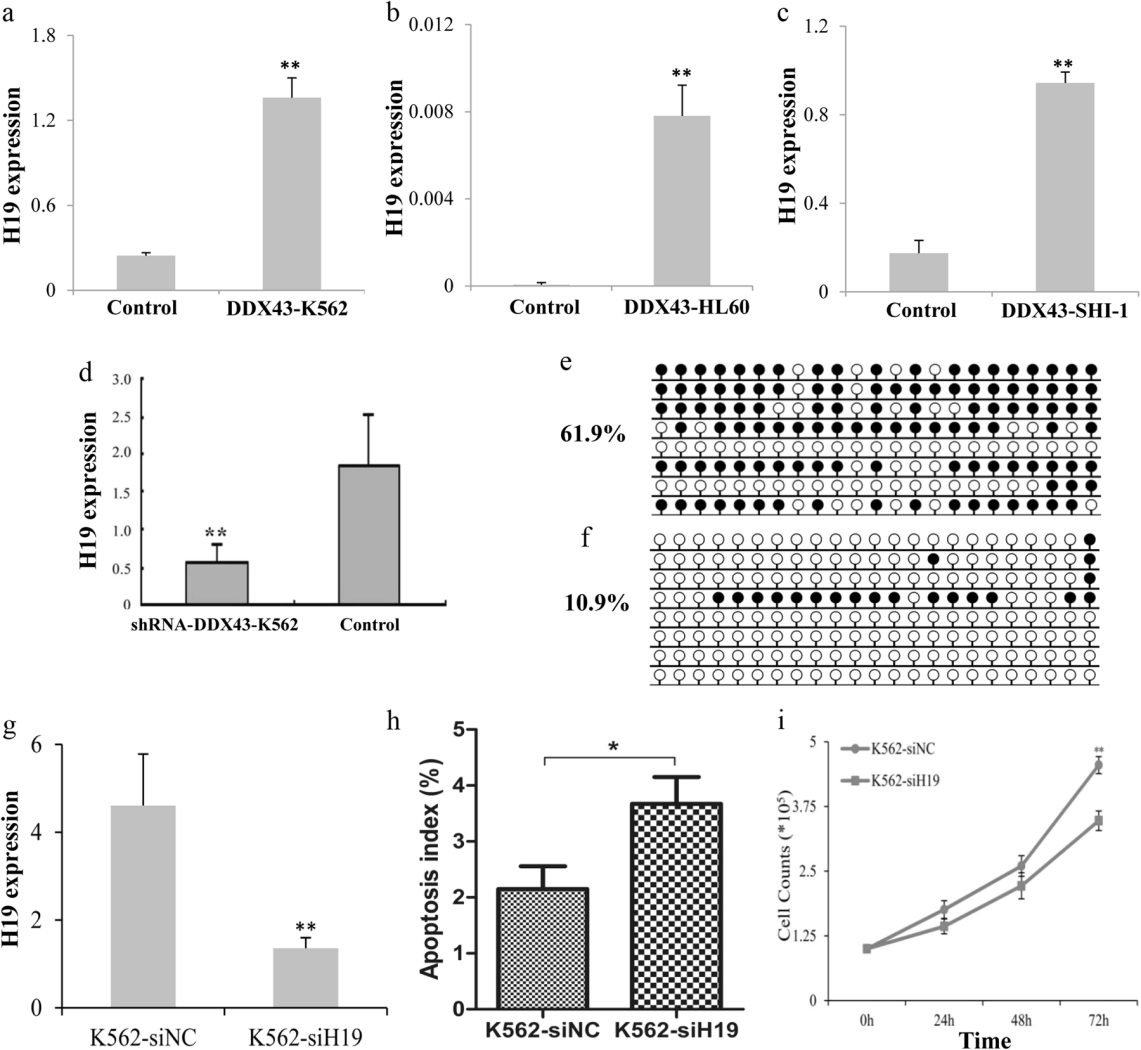
*DDX43* promoted H19 expression by demethylation. **a**–**d** Expression of H19 in *DDX43*-transfected K562, HL60, SHI-1 and *DDX43-*silencing K562 cells was detected by RQ-PCR. **e**, **f** Promoter methylation levels of H19 in control vector or *DDX43* transfected cells were detected by bisulfite sequencing. **g** Expression of H19 was detected by RQ-PCR. **h** K562 cells transfected with H19 siRNA or a control was used to apoptotic assays in serum-free media for 48 h. Cells were subjected to staining, followed by flow cytometry analysis. **i** K562 cells transfected with H19 siRNA or a control were subjected to proliferation assays in 10% FBS media and the live cells were counted at 24, 48, and 72 h. *P < 0.05; **P < 0.01. Error bars indicate SD (*n* = 3)

To explore the molecular mechanism of the *DDX43* effects, we performed deep sequencing assay to identify related genes for *DDX43*. A number of genes and microRNAs were up- or down- regulated, including H19 (Table 1). Our study found that the H19 expression increased in *DDX43*-transfected K562, HL60, and SHI-1 cells (Fig. 5a–c), and decreased after *DDX43* silencing (Fig. 5d), suggesting *DDX43* could increase H19 expression. Furthermore, the methylation level of H19 promoter was identified to be decreased in *DDX43*-transfected cells (Fig. 5e, f). Moreover, clinical sample analysis found H19 overexpression was associated with promoter hypomethylation and was upregulated during CML progression [26]. These results indicated that *DDX43* may regulate the expression of H19 through demethylation.

**Table. 1:** Upregulated and downregulated genes in *DDX43*-K562 by deep sequencing assay

20 upregulated genes	DDX43-K562/Control	20 downregulated genes	DDX43-K562/Control
*DDX43*	432.5745	*EPB41L3*	0.001285
*H19*	91.57027	*MGST1*	0.006269
*ITGBL1*	83.46942	*PPFIBP2*	0.006
*PPARGC1A*	53.39036	*SPARC*	0.017029
*SPATA17*	34.96689	*ARHGAP15*	0.010071
*LAPTM5*	20.31337	TUBA3C	0.008135
*SPANXA2*	19.73602	*FAM5C*	0.022559
*KIF1A*	17.12457	*STXBP6*	0.020508
*GPRC5C*	13.10999	*PRDM9*	0.02692
*PDE2A*	12.78386	*NXF2*	0.025782
*LPL*	11.50522	*PASD1*	0.02479
*RBPMS2*	10.95067	*TDRG1*	0.030693
*COL1A2*	10.80316	*NXF2B*	0.029298
*TMEM163*	9.399711	*C6orf145*	0.028922
*SHANK1*	8.798151	*TBX15*	0.028922
*HLA-DOB*	7.444569	*C9orf125*	0.033839
*ANKRD62*	7.37238	*MAML3*	0.039916
*TTYH3*	6.834143	*ADAM19*	0.039347
*XKR8*	6.821518	*MALT1*	0.045117
*RASGRP2*	6.69587	*GNG11*	0.044672

Many *DDX43* related genes were involved in tumor growth, including H19. To explore the role of H19 in leukemia, the siRNA of H19 was transfected into K562 cells (K562-siH19). Expression of H19 was detected by RQ-PCR and decreased H19 was found in H19 siRNA-transfected cells, compared with control (Fig. 5g). Apoptosis was assayed for K562 cells transfected with H19 siRNA or a control in serum-free media for 48 h. K562-siH19 showed increased apoptosis rate compared with control (Fig. 5h). Moreover, K562-siH19 cells achieved reduced proliferation, compared to the control group (Fig. 5i). These results suggested that silencing H19 could promote K562 apoptosis and inhibit proliferation.

### Arresting of miR-186 by DDX43 and miR-186 mediates K562 functions

**Fig. 6:**
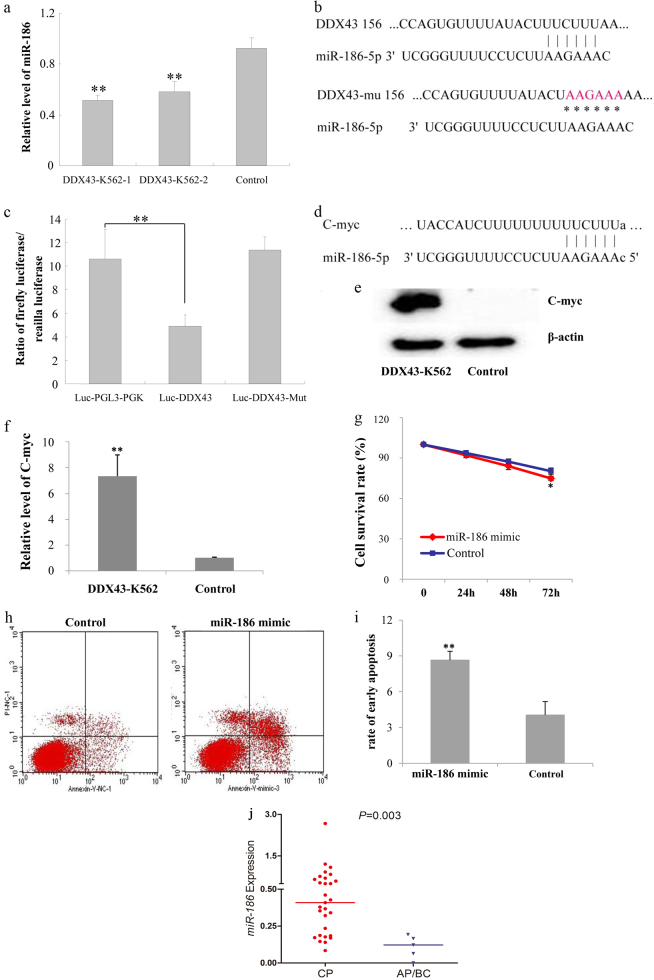
miR-186-mediated *DDX43* functions. **a** Expression of miR-186 in *DDX43* or control vector transfected K562 cells was detected by RQ-PCR. **b** Binding of miR-186 to *DDX43* 3′ UTR was detected by luciferase assay. **c** The wild-type (Luc-*DDX43*) and mutant (Luc-*DDX43*-Mut) 3′ UTR sequences of *DDX43* for luciferase activity assays. **d** Bioinformatics analysis showed the binding of miR-186 to c-Myc 3′ UTR. **e**, **f** Expression of c-Myc in *DDX43-* or control vector-transfected cells was detected by western blot and RQ-PCR. **g** K562 cells transfected with miR-186 mimic or control vector were cultured in serum-free media. Cells were harvested and surviving cells were counted. **h**, **i** K562 cells transfected with miR-186 mimic or a control were harvested for apoptotic assays in serum-free media after 48 h. Cells were subjected to staining, followed by flow cytometry analysis. **j** Relative expression level of miR-186 in CP and AP/BC stage of CML patients was presented with scatter plots. The median level of miR-186 in each group was shown with horizontal line. *P < 0.05; **P < 0.01. Error bars indicate SD (*n* = 3)

The deep sequencing also identified that miR-186 was downregulated in *DDX43*-transfected K562 cells (Table 2). RQ-PCR results further confirmed the decreased miR-186 expression in *DDX43*-transfected cells (Fig. 6a), suggesting that *DDX43* may be involved in the reverse regulation to miR-186.

**Table. 2:** Upregulated and downregulated microRNAs in *DDX43*-K562 by deep sequencing assay

20 upregulated microRNAs	DDX43-K562/Control	20 downregulated microRNAs	DDX43-K562/Control
miR-218-5p	396.223	miR-1268a	0.001539
miR-520	131.4816	miR-151a-5p	0.002198
miR-103a-3p	107.1052	hsa-miR-4429	0.012104
miR-107	107.1052	miR-135b-5p	0.020591
miR-9-3p	74.74683	miR-34c-5p	0.044525
miR-517c-3p	65.74081	miR-3180	0.060846
miR-373-3p	48.38775	miR-4301	0.074321
miR-520b	47.69739	miR-320c	0.084726
miR-526b-3p	47.69739	miR-320b	0.084726
miR-520e	47.69739	miR-22-3p	0.085581
miR-522-3p	47.06979	miR-3180-3p	0.091131
miR-3529-3p	43.59387	miR-1285-3p	0.092263
miR-3180-5p	37.18514	miR-4448	0.09414
miR-1972	29.81087	miR-30c-5p	0.129583
miR-1255b-5p	26.35908	miR-151a-3p	0.134717
miR-518d-5p	22.5935	miR-499a-5p	0.150078
miR-520c-5p	22.5935	miR-32-5p	0.294186
miR-526a	22.5935	miR-1254	0.313799
miR-518f-5p	22.5935	miR-186-5p	0.362075
miR-1304-5p	16.47443	miR-10b-5p	0.362075

Bioinformatics analysis showed that miR-186 could bind to *DDX43* 3′ UTR. In order to confirm this result, the 3′ UTR of *DDX43* was cloned to the downstream of luciferase reporter gene. Luciferase activity assay showed that the luciferase relative expression of the group co-transfected with *DDX43*-3′ UTR and miR-186 was lower than the control groups (PGL3-PGK + miR-186, negative control; *DDX43*-3′ UTR MUT + miR-186, negative control) (Fig. 6b, c). The results supported the direct binding of miR-186 to *DDX43* 3′ UTR region, and *DDX43* as a potential target of miR-186. Combining with the effect of *DDX43* on miR-186 expression, we speculate that *DDX43* may be involved in the reverse regulation of miR-186. Further study is needed to determine the detailed dual-regulatory mechanisms. Moreover, bioinformatics analysis showed that c-Myc was another target of miR-186 (Fig. 6d). Our results identified that c-Myc increased in *DDX43*-transfected cells (Fig. 6e, f).

We transfected miR-186 mimic into K562 cells, and analyzed the role of miR-186 on K562 cells. The survival rate of miR-186-K562 cell was decreased (Fig. 6g) and the apoptosis rate was increased (Fig. 6h, i), compared to the control group, suggesting that miR-186 could inhibit K562 survival and promote apoptosis. In addition, the level of miR-186 in CML patients at AP/BC stages (median 0.12, range 0–0.19) was lower than that at the CP stage (median 0.41, range 0.08–2.66) (*P* = 0.003) (Fig. 6j).

## Discussion

Our previous study confirmed that *DDX43* overexpression, regulated by the hypomethylation of its promoter, was associated with the progression of CML. In this study, we found that *DDX43* enhanced survival and colony formation, inhibited cell apoptosis, promoted tumorigenesis, and CML progression in vitro and in vivo.

To elucidate the molecular mechanisms of *DDX43* in leukemia, the deep sequencing assay was performed to screen related genes for *DDX43*. Various mRNAs related to *DDX43* were up- or down- regulated. H19, RASGRP2, ITGBL1, and PPARGC1A were upregulated, the roles of which are identified in tumorigenesis, cell growth, cell adhesion, and nucleotide binding; MGST1, SPARC, and FAM5C were downregulated, whose functions are involved in the response to drug, cell proliferation, cell cycle and so on. At the same time, some microRNAs related with *DDX43* were up- or down- regulated. Some tumor-promoting microRNAs, such as miR-218-5p, miR-107, and miR-9-3p, were upregulated; some tumor-suppressing microRNAs, such as miR-186, miR-22-3p, and miR-1285-3p, were downregulated. We confirmed that H19 expression was increased and miR-186 expression was decreased in *DDX43*-transfected cells using RQ-PCR. Other genes and microRNAs changed in *DDX43*-transfected cells might be studied in a future study.

We further showed that *DDX43* induced H19 promoter demethylation, and then increased its expression. H19 has been revealed to be upregulated in various carcinomas and to play important roles in the occurrence, development, metastasis, and prognosis of tumors [27–30]. Our previous study indicated that H19 expression, associated with its promoter methylation, was significantly upregulated in CML patients, and its expression increased as disease progressed [26]. H19 acts mainly through two ways: first, miR-675, encoded by the first exon of H19, regulates the relevant tumor suppressor genes [28, 31–33]; second, the total length of H19 combines with microRNAs or related proteins to regulate their functions [28, 34, 35]. We also detected miR-675 expression in *DDX43-* transfected K562, HL60, and SHI-1 cells. However, miR-675 was downregulated in *DDX43*-transfected K562 cells, upregulated in *DDX43*-transfected SHI-1 cells and showed no difference in *DDX43*-transfected HL60 cells. It is suggested that miR-675 regulation might not be the main factor in leukemic cell function.

In addition, we also focused on miR-186 which was decreased in *DDX43*-transfected cells. Luciferase activity test showed that miR-186 might bind to the *DDX43* 3′ UTR region, and *DDX43* may be a potential target gene of miR-186. We also detected the effect of miR-186 on K562 cells. Our results suggested that miR-186 could inhibit K562 survival and promote apoptosis. Recently, miR-186 has been considered as a tumor suppressive microRNA in many solid cancers, such as prostate cancer and non-small cell lung cancer (NSCLC) [21, 36, 37]. However, it was also reported that miR-186 acted as an oncogenic microRNA by modulating PTTG1 expression and accelerating migration and invasion in human NSCLC cells [38]. As to hematologic malignancies, Ferrer G identified the downregulation of miR-186 expression in chronic lymphocytic leukemia [39]. Our group previously identified that miR-186 transcript frequently decreased in AML. Moreover, in this study, miR-186 decreased in AP/BC stage compared with the CP stage in CML, suggesting that miR-186 downregulation may promote the progression of CML. Downregulated expression of miR-186 also increased the expression of c-Myc. We speculate that it may involve complex regulatory mechanisms that need further study.

Now, we will propose the following hypothesis: miR-186 expression is downregulated in the progression of CML, leading to the upregulation of target gene—*DDX43* in CML; *DDX43* further upregulates the expression of H19 through demethylation. In summary, the signal of miR-186/*DDX43*/H19 may be involved in regulating the function of leukemia cells.

In conclusion, our results indicate that *DDX43* enhances survival and colony formation, inhibits cell apoptosis, and promotes tumorigenesis and CML progression. The potential mechanism involves the arresting of miR-186 and releasing of H19 by *DDX43*.

## Materials and methods

### Construct generation

A *DDX43*-expressing construct was designed by our laboratory. Briefly, a cDNA sequence containing *DDX43* was inserted into a lenti-virus vector containing green fluorescent protein in the restriction enzyme site *Bam*HI.

### RNA isolation, reverse transcription, and RQ-PCR

Total RNA was isolated (mirVana miRNA Isolation Kit, Ambion, USA) and then reverse transcribed into cDNA. RQ-PCR was performed as described previously [40]. *DDX43* was amplified with primers of 5′-CCTTTCAATGTTATCCTGAG-3′ (forward) and 5′-TATTCTTCAGATTGACGAAG-3′ (reverse). The specific forward primer of miR-186 was 5′-CAAAGAATTCTCCTTTTGGGCT-3′, while H19 was amplified with primers of 5′-GGGTCAGACAGGGACATGG-3′ (forward) and 5′-GAGCGGTGAGGGCATACA-3′ (reverse). MiR-186 and H19 expressions were determined as reported previously [21, 41].

### DNA isolation, modification, and methylation detection

DNA extraction and modification were performed as described previously [8]. The level and density of H19 promoter methylation were detected using real-time quantitative MSP and bisulfite sequencing PCR as previously reported [41].

### Cell survival and apoptosis assays

To analyze cell survival, 1×10^6^ K562 or Meg-01cells (ATCC, Manassas, USA) were cultured in 25 cm^2^ tissue culture flasks for 72 h in Iscove’s Modified Dulbecco’s Medium (IMDM, Wisent) without FBS (ExCell Bio). The viable cells were numbered using trypan blue exclusion. To study cell apoptosis, 1×10^6^ cells were cultured in 25 cm^2^ tissue culture flasks for 48 h in IMDM without FBS. The cultured cells were also stained for apoptosis assay using the apoptosis detection Kit (Annexin V-FITC, BD, 556547; or Annexin V-PE/7AAD, BD, 559763), followed by flow cytometry analysis.

### Colony formation

Soft agarose assay: 2% agarose was plated on six-well plates, and then 1×10^3^ cells were inoculated into 0.3% agarose containing 10% FBS. Colonies were numbered and photographed after 4 weeks. Methylcellulose assay: 1×10^3^ cells in IMDM were mixed in 1% methylcellulose supplemented with 10% FBS and plated on six-well tissue culture plates. Colonies were numbered and photographed 2 weeks after cell inoculation.

### Tumorigenesis assays in nude mice

The animal protocol was approved by the Animal Care Committee of the Affiliated People’s Hospital of Jiangsu University. The *DDX43*- or mock-transfected K562 cells were injected randomly into 5-week-old CD1 strain nude mice subcutaneously (1.5×10^7^ cells per mouse, seven mice per group). Tumor growth was assessed every 4 days. The mice were killed and the tumors were removed at day 30 post inoculation as described previously [42]. Tumors were stored in −80 °C for PQ-PCR and western blot to detect *DDX43* expression. *DDX43* shRNA- or a control vector-transfected *DDX43*-K562 cells were injected randomly into CD1 strain nude mice subcutaneously. Eight mice were included in each group. Mice were photographed and killed after 4 weeks of injection. Tumor sizes were measured.

### CML progression in SCID mice

In the CML progression assay, the *DDX43*- or mock-transfected K562 cells were injected into 5-week-old SCID mice randomly via tail vein (1.5×10^7^ cells per mouse, five mice per group). Mouse weight was monitored weekly. The peripheral WBC was counted weekly. When the mice suffered from CML symptoms (4 weeks after injection), they were killed and bone marrows were obtained to detect blasts with Wright−Giemsa stain.

### Transfection of tumor cells with shRNA or siRNA

The siRNAs and shRNAs were designed and synthesized (GenePharma, Shanghai, China). Three shRNAs targeting *DDX43* expression and one negative control were used in this experiment: shRNA-1 (*DDX43*-homo-909 GCAGATTTACCACCAATTAAG); shRNA-2 (*DDX43*-homo-1066 GCACATTTGATGACGCCTTTC); shRNA-3 (*DDX43*-homo-1175 GCAAGGAATAGATCTTATAGG); and negative control (TTCTCCGAACGTGTCACGT). SiH19-1977 (CCCGUCCCUUCUGAAUUUATT) and H19 siRNA-negative control (UUCUCCGAACGUGUCACGUTT) were used for H19-silencing experiments. ShRNA or siRNA transfection was performed using Lipofectamine 2000 (Invitrogen). Western blot and RQ-PCR were carried out 72 h after transfection.

### Western blot analysis

Cells were lysed with 100 µl of lysis buffer containing protease inhibitors (Beyotime, Shanghai, China). The lysates were subjected to sodium dodecyl sulfate-polyacrylamide gel electrophoresis (SDS-PAGE), transferred to a polyvinylidene fluoride (PVDF) membrane, and then incubated with an anti-*DDX43* primary antibody (H00055510-M07, Abnova) overnight at 4 °C. Horseradish peroxidase (HRP)-conjugated goat-anti-mouse secondary antibody (sc-2005, Santa Cruz Biotechnology) was used. The protein bands were detected by enhanced chemiluminescence detection. Anti-β-actin antibody (AA128, Beyotime, Shanghai, China) was used as a loading control.

### Luciferase activity assay

Luciferase activity assays were carried out (Promega Luciferase Assay System, USA). The vector was purchased from HuiJun (Guangzhou, China). The wild-type (Luc-*DDX43*) or mutant (Luc-*DDX43*-Mut) sequences of *DDX43* 3′ UTR was inserted into a dual luciferase reporter vector (Promega, USA). A total of 5×10^4^ HepG2 cells were cultured in Dulbecco's Modified Eagle Medium (DMEM) containing 10% FBS in 24-well plates. The cells were co-transfected with 0.8 μg of Luc-PGL3-PGK or Luc-*DDX43* or Luc-*DDX43*-Mut and 24 pmol of miR-186 mimic or scramble miR mimic, and the pRL-TK plasmid for internal normalization using Lipofectamine 2000. We measured Firefly and Renilla luciferase activities immediately using dual-luciferase assays (Promega, USA) 24 h after transfection.

### Patients

This study was approved by Institutional Ethics Committee of the Affiliated People’s Hospital of Jiangsu University. Bone marrow samples from 34 CML patients (29 cases at CP stage, 3 at AP stage, and 2 at BC stage) were obtained with written informed consent. Bone marrow samples were enriched for mononuclear cells using density gradient centrifugation.

### Statistical analysis

All assays were performed at least in triplicate. Statistical analyses were performed as previously described [25, 43]. In brief, results expressed as mean values ± SD of all experiments were used for statistical analysis. Student’s *t* test or one-way analysis of variance was carried out to compare the difference among two or multiple groups. The level of significance was set at *P* < 0.05.
